# Monthly Record of Deaths in the City of Buffalo

**Published:** 1854-06

**Authors:** Jas. M Newman

**Affiliations:** Health Physician


### ART. V.—*Record of Mortality in the City of Buffalo, for the Month of April,* 1854. By J. M. Newman, Health Physician.

| DISEASES. | No. | Males. | Females. |
|---|---|---|---|
| Accidens, | 2 | 2 | |
| Apoplexia, | 1 | 1 | |
| Asphyxia Immersorum, | 10 | 4 | 1 |
| Asthma, | 1 | | 1 |
| Cancer Degener Hepaticus, | 1 | 1 | |
| Cynanche Trachealis, | 3 | | 3 |
| Debilitatis, | 6 | 4 | 2 |
| Diarrhœa, | 1 | | |
| Dysenteria, | 1 | | 1 |
| Eclampsia, | 10 | 4 | 4 |
| Enteritis, | 1 | | 1 |
| Erysipelas, | 1 | | 1 |
| Febris Typhus, | 3 | 3 | |
| " Typhoid, | 3 | 1 | 2 |
| " Biliera, | 1 | | 1 |
| " Scarlatina, | 3 | 1 | 1 |
| Hydrops, | 2 | | 2 |
| " Abdominis, | 1 | 1 | |
| " Cerebri, | 3 | 3 | |
| " Pectoris, | 1 | 1 | |
| Hyperæmia Pulmonalis, | 2 | 1 | 1 |
| Ignotus, | 28 | 11 | 11 |
| Influenza, | 2 | 1 | 1 |
| Intemperantia et Delir. Tremens, | 4 | 4 | |
| Marasmus, | 3 | 2 | 1 |
| Morbus Renis et Hepaticus, | 1 | | 1 |
| " Brightii, | 1 | 1 | |
| Mortificatio, | 1 | 1 | |
| Natus Mortuus, | 10 | | |
| Ossificatio Valvularum Cordis, | 1 | 1 | |
| Pertussis, | 3 | 2 | 1 |
| Phrenitis. | 1 | 1 | |
| Phthisis Pulmonalis, | 22 | 15 | 7 |
| Pneumonia, | 12 | 7 | 5 |
| Prostatitis, | 1 | 1 | |
| Rubeola, | 11 | 3 | 6 |
| Scrophula, | 1 | 1 | |
| Senectus, | 5 | 2 | 3 |
| Variola, | 2 | | 2 |
| | 166 | | |

| | |
|---|---|
| Males, | 80 |
| Females, | 59 |
| Sex not given, | 27 |
| Total, | 166 |

AGES.

| | | | |
|---|---|---|---|
| Still-born, | 10 | Between 12 years and 20 years, | 3 |
| 1 day, | 2 | " 20 " " 30 " | 6 |
| Between 1 day and 15 days, | 9 | " 30 " " 40 " | 13 |
| " 15 days and 30 days, | 5 | " 40 " " 50 " | 7 |
| " 1 month and 3 months, | 6 | " 50 " " 60 " | 8 |
| " 3 months and 6 months, | 7 | " 60 " " 70 " | 3 |
| " 6 " " 9 " | 11 | " 70 " " 80 " | 10 |
| " 9 " " 12 " | 6 | " 80 " " 90 " | 1 |
| " 1 year and 3 years, | 28 | " 90 " "100 " | 2 |
| " 3 years and 6 years, | 9 | Ages not given, | 7 |
| " 6 " " 12 " | 13 | | |
| Total, | | | 166 |

NATIVITY.

| | | | |
|---|---|---|---|
| American, | 73 | Hollander, | 3 |
| English, | 2 | Welch, | 1 |
| Scotch, | 1 | Canadian, | 2 |
| Irish, | 10 | Colored, | 2 |
| German, | 39 | Country not given, | 42 |
| French, | 1 | | |
| Total, | | | 166 |

